# The Antimicrobial Effect of Thymol and Carvacrol in Combination with Organic Acids Against Foodborne Pathogens in Chicken and Beef Meat Fillets

**DOI:** 10.3390/microorganisms13010182

**Published:** 2025-01-16

**Authors:** Ioanna Mantzourani, Maria Daoutidou, Athanasios Alexopoulos

**Affiliations:** 1 Laboratory of Food Processing, Faculty of Agriculture Development, Democritus University of Thrace, 68200 Orestiada, Greece; mdaoutid@agro.duth.gr; 2 Laboratory of Microbiology, Biotechnology & Hygiene, Faculty of Agriculture Development, Democritus University of Thrace, 68200 Orestiada, Greece; alexopo@agro.duth.gr

**Keywords:** carvacrol, thymol, chicken fillets, beef fillets, organic acids, antimicrobial activity, antioxidant activity

## Abstract

Bioactive compounds and organic acids are applied to a wide range of foods against different types of foodborne pathogens. In the present study, carvacrol and thymol (1000 mg/L) were applied in wine-based marinades, alone or in combination with them and in combination with tartaric acid, malic acid, ascorbic acid, citric acid, and acetic acid (in concentration 0.1% *w*/*v*), in chicken and beef fillets and their antimicrobial activity, antioxidant capacity, and pH were estimated during refrigerated storage. Likewise, their antimicrobial activity was recorded against *Enterobacteriaceae*, total mesophilic bacteria, yeasts/molds, and lactic acid bacteria. The outcome demonstrated that both meats kept under similar storage conditions (4 °C/9 days) exhibited lower microbial growth, particularly with *Enterobacteriaceae,* when treated with wine-based carvacrol—thymol marinades and may extend their shelf-life. This antimicrobial action was more pronounced in the beef samples. The total phenolic content (TPC) and the antioxidant activity of the applied marinades were determined using the Folin−Ciocalteau method and ABTS and DPPH radical scavenging activity methods, respectively. The results revealed that marinades with thymol and/or carvacrol in combination with acetic or ascorbic acid had greater TPC and antioxidant activity. The pH values of the respective marinades applied to both chicken and beef fillets exhibited an upturn during storage. Consequently, these marinades, even at low concentrations, could be used as natural preservatives in meat products.

## 1. Introduction

Foodborne diseases are continuously affecting consumers, food and health authorities, and the food industry. Such diseases are a major cause of human morbidity and mortality, responsible for 600 million illnesses, 420,000 deaths, and 33 million disability-adjusted life years in 2010 [1]. On the other hand, meat products are among the most perishable, with more than 20% of world meat production valued at $150 billion, lost or wasted due to microbial spoilage [2]. In particular, the abundance of foodborne pathogens appears to be in fresh meat and meat products. Therefore, alternative technologies for long-term preservation, quality assurance, and safety of meat are constantly being pursued by the food industry [3].

Nitrites are used in meat products as food additives to combat pathogenic bacteria, such as *Clostridium botulinum* and *Listeria monocytogenes*, and to reduce lipid oxidation [3]. However, nitrites have been proven to exhibit carcinogenic activity, and for this reason, contemporary research has focused on providing consumers with natural additives with no or limited potential health hazards associated with their consumption [4]. The aim of preservation methods is to inhibit microbial spoilage and minimize oxidation and enzymatic spoilage. Traditional methods of meat preservation include drying, smoking, brining, and canning, which have been replaced by new preservation techniques, such as chemical, bio preservative, and nonthermal techniques, over the years [5]. However, the main trend for meat preservation is the use of biopreservatives [6]. Phenolic antioxidants have extensive use with the intention to delay, retard, or prevent the negative effects of lipid oxidation, the main cause of meat spoilage [7]. Phenolic compounds appear in large amounts in plants and their products. Naturally occurring phenolic compounds are considered promising substances by the meat industry for their antioxidant and antimicrobial properties, while they are generally recognized as safe (GRAS) [8].

Furthermore, chicken meat remains attractive for consumption given its high nutritive value, high proportion of unsaturated fatty acids, low energy content, and fewer associated religious restrictions [9,10]. On the other hand, beef meat is mostly consumed due to its flavor and the complex composition of macronutrients, the balanced composition of amino acids, vitamins from the B group, and high contents of iron, zinc, and phosphorus [11,12]. Physicochemical (animal species, age, type of nutrition, sex, and muscle type) and biochemical parameters (myofibrillar proteins, connective tissue proteins, and collagen) affect the tenderness of beef, which is a basic factor for consumer acceptance [13,14].

Marination is commonly used to enhance the functional and sensory properties of meat by soaking, injecting, or tumbling it with aqueous solutions composed of different ingredients. The most commonly available are water emulsion marinades, which consist of salt, sugar, vinegar, citric acid, and other supplements like spices [15]. This kind of marinade has the ability to increase water retention and modify the pH. The use of acidic marinades not only leads to an optimum water retention capacity, but also to tenderization of the meat [16].

Essential oils have received great interest because of their low toxicity, pharmacological activity, and economic viability [17]. As reported by the study of Martuccia et. al. [18], the antibacterial activity of the essential oil from dried leaves of oregano in minimum inhibitory concentration was yielded against *Escherichia coli* strains (MICOEO = 1600–1800 ppm) and *Staphylococcus aureus* strains (MICOEO = 800–900 ppm). These bacteria appear to be among the main factors for food spoilage, and for this reason, these MICs were satisfactory. In another study by Hossain et al. [19] eight essential oils (EOs) of plant origin, namely, eucalyptus, tea tree, basil, oregano, cinnamon, mandarin, peppermint, and thyme essential oil were evaluated for their ability to inhibit the growth of *Aspergillus niger*, *Penicillium chrysogenum*, *Aspergillus flavus*, and *Aspergillus parasiticus* and it was revealed that a combined formulation of oregano with thyme essential oil resulted in a synergistic effect against *A. flavus*, *A. parasiticus* and *P. chrysogenum*. In addition, a combination of oregano and thyme, cinnamon and thyme, and oregano and mint essential oils was used against *A. flavus,* and similar synergistic effects were recorded. Therefore, it becomes obvious that the combination of some particular essential oils results in synergism as a result of the combined activities of two or more constituents of essential oils. Furthermore, it can be stated that pathogens cannot easily acquire resistance to multiple components of two or more essential oils.

The two major components of oregano and thyme essential oils that exhibit strong antimicrobial activity are thymol and carvacrol. The use of thymol and carvacrol would be ideal for food at low concentrations because of their compatibility with several food matrices and their ability to not negatively alter the sensorial profile of the food applied. Several studies have focused on the synergistic effect of thymol and carvacrol in combination [20]. Both thymol and carvacrol are able to destroy the outer membrane of bacteria and increase its fluidity [21,22]. From the literature, it is known that whole essential oils have greater antimicrobial activity than the major components, so the addition of small amounts of other organic acids may enhance their efficacy in real food systems. This addition is a way to balance sensory acceptance and antimicrobial activity [23]. Major compounds have less impact on the odor and flavor of foods in comparison with the whole essential oil [24].

The antimicrobial activity can be enhanced at the junction of bioactive substances and organic acids [20]. Weak organic acids have been of considerable value as food preservatives since they are also food ingredients and are often naturally produced by microorganisms [25]. More specifically, acetic and citric acids are used in pickles, salads, dressings, and sauces, while ascorbic acid is used as a natural preservative in juices and marinades. Tartaric acid is present mainly in wines and vinegar, and malic acid is the initial substrate for fermentation in wines or juices. Both have been used to control food spoilage in several food systems [25,26].

The purpose of this paper was to study the antimicrobial and antioxidant activities of thymol and carvacrol, in combination with these organic acids, in wine-based marinades applied to chicken and beef fillets during refrigeration storage. To our knowledge, no research study has been conducted regarding these combinations of organic acids with carvacrol and/or thymol in marinades and their subsequent antioxidant and antimicrobial activities in both chicken and beef fillets.

## 2. Materials and Methods

### 2.1. Raw Materials

The chicken and beef fillets used for marination were purchased from the local market of Nea Orestiada in the Evros region of Greece.

The red wine (merlot cabernet, 12.5% ethanol) used for the marination of the chicken and beef fillets was produced in northeastern Greece by local wine producers using the traditional method of vinification, and it is not a standardized product.

### 2.2. Chemicals

The bioactive substances carvacrol (CID 10364) and thymol (CID 6989), as well as the acids tartaric (CID 875), malic (CID 525), ascorbic (CID54670067), acetic (176), and citric (CID 311), were purchased from Sigma-Aldrich (Saint Louis, MO, USA).

### 2.3. Preparation and Marination of Chicken and Beef Fillets

After the fillets were purchased from the local market within 24 h of slaughter, they were immediately transferred to the laboratory. The samples were cut under sterile conditions with a flame-sterilized kitchen knife into portions of 50 g, placed in sterile plastic containers, and stored under refrigeration conditions (4 °C) until marination (less than 1 h). The marinade consisted of the following ingredients (% *w*/*v*): red wine (12.5), salt (1.5), tartaric acid (0.1), malic acid (0.1), ascorbic acid (0.1), acetic acid (0.1), and citric acid (0.1) in different combinations between thymol (1000 mg/L) and/or carvacrol (1000 mg/L) and organic acids. Chicken and beef fillets were immersed in the marinade and mixed with sterile forceps under sterile conditions. Marinated meat samples (in triplicate) were then covered and stored at 4 °C.

### 2.4. Microbiological Analysis of Meat Fillets Portions

The microorganisms mostly responsible for the spoilage of meat fillets (*Enterobacteriaceae,* Total Mesophilic Bacteria (TMB), and yeasts and molds) and their relevant populations during refrigerated storage were identified. In addition, the levels of LAB were also examined. Therefore, representative samples of 10 g of meat fillets were taken from the inside on the 1st, 3rd, 5th, and 7th day, and from unspoiled samples on the 9th and 11th day of refrigerated storage, and they were mixed with 90 mL of sterile 1/4 Ringers solution (Sigma Aldrich). Afterwards, the mixture was subjected to decimal serial dilutions. As in previous studies [27], the following tests were performed: (i) total aerobic counts on plate count agar (Oxoid Ltd., Basingstoke, UK) at 30 °C for 48 h, (ii) lactobacilli [Gram (+), catalase (−)] on acidified MRS agar (Oxoid Ltd., Basingstoke, UK) at 37 °C for 48 h anaerobically (Anaerobic jar, Anerocult C, Merck), (iii) Enterobacteria on violet red bile glucose agar (Oxoid Ltd., Basingstoke, UK) at 37 °C for 24 h, and (iv) yeasts and molds on malt agar (Oxoid Ltd., Basingstoke, UK) at 30 °C for 48 h. The results from the culture plates (containing between 30 and 300 colonies) are presented as the logarithm of the mean colony-forming units (Log_10_ CFU/g meat fillet).

### 2.5. pH Analysis

The pH value of each marinade just before the application to meat was determined using a digital pH meter (Mi 150 pH/Temperature Bench Meter, Milwaukee Electronics Kft, Szeged, Hungary). More specifically, 10 mL of each marinade treated sample was measured at room temperature on the 1st, 3rd, 5th, and 7th days, and in unspoiled samples on the 9th and 11th days of refrigerated storage.

### 2.6. Antioxidant Activity Analysis

#### 2.6.1. DPPH—Scavenging Ability Method

For the determination of antioxidant activity, the DPPH scavenging ability method was conducted as follows: 3 mL of DPPH (2,2 Diphenyl-1-picrylhydrazyl) solution (60 μM) and 30 μL of each marinade sample were added into a cuvette after 10-fold dilution of each sample. As a blank sample, 3 mL of DPPH solution and 30 μL of water were added to a cuvette. The samples and blank sample were then placed in a shaded space and incubated for 30 min at room temperature. Finally, the absorbance was detected at 515 nm on a photometer [28]. The results are presented as μg Trolox Eq/mL marinade.

#### 2.6.2. ABTS—Scavenging Ability Method

For the determination of antioxidant activity, the ABTS scavenging ability method was used. More specifically, 3 mL of ABTS [2,2 azinobis-(3-ethylbenzothiaziline-6-sulfonate)] reagent and 30 μL of each sample were placed into a cuvette after 10-fold dilution of each sample. As a blank sample, 3 mL of ABTS and 30 μL of ethanol were placed in the cube. All the samples were incubated for 6 min at 30 °C in a shaded place. Finally, the absorbance was detected at 734 nm on a photometer [29]. The results are presented as μg Trolox Eq/mL marinade.

#### 2.6.3. TPC—Total Phenolic Content

The determination of the total phenolic content by the Folin−Ciocalteau Micro Method was conducted as follows: First, 20 μL of each sample, after 10-fold dilution, was added in a cuvette with 100 μL of Folin−Ciocalteau reagent and 1.58 mL of water. The cuvettes were placed in a shaded space for 4 min at room temperature, and then 300 μL of sodium carbonate solution (Na_2_CO_3_ 20%) was added. The samples were then incubated for 30 min at 40 °C. Finally, absorbance was measured at 765 nm [30]. The results are presented as mg gallic acid Eq/mL marinade.

### 2.7. Planning and Analysis Overview

For the marinades, an experimental design was used that combined carvacrol, thymol, and acids (tartaric, ascorbic, citric, acetic, and malic acid), thus obtaining the following 15 combinations: 1. Wine Salt, 2. Wine Salt Carvacrol, 3. Wine Salt Thymol, 4. Wine Salt Carvacrol/Thymol, 5. Wine Salt Carvacrol Tartaric acid, 6. Wine Salt Thymol Tartaric acid, 7. Wine Salt Tartaric acid, 8. Wine Salt Carvacrol Malic acid, 9. Wine Salt Thymol Malic acid, 10. Wine Malic acid, 11. Wine Salt Carvacrol Ascorbic acid, 12. Wine Salt Thymol Ascorbic acid, 13. Wine salt Ascorbic acid, 14. Wine Salt Carvacrol Acetic acid, 15. Wine Salt Citric acid. 

The marinades were prepared in adequate quantities. Initially, the pH, total phenolic content, ABTS, and DPPH scavenging abilities of the marinades were examined in triplicate. Accordingly, multiple 50 g portions of marinated and unmarinated (controls) chicken and beef fillets were stored at 4 °C until any evidence of spoilage (odor, color, texture). The pH value, enterobacteria, total aerobic counts, yeast and molds, and lactobacilli from triplicate marinated and control meat fillets were determined on the 1st, 3rd, 5th, 7th, 9th, and 11th days or until spoilage. In total, 45 chicken samples (+3 controls) and 45 beef samples (+3 controls) were evaluated for their microbiological quality. Most samples were spoiled after the 9th day of storage, and only the chicken fillets marinaded with wine, salt, and acetic or citric acid lasted for 11 days. The number and types of samples are presented in Table S1 (Supplementary Materials). 

### 2.8. Statistical Analysis

Bacterial counts were logarithmically transformed (normalization) and presented as Log CFU/g of meat fillet. For comparison, wine treatment was considered as the control, and bacterial counts from other treatments were compared using analysis of variance with Dunnett’s *post hoc* application following a variance check. A comparison of the total phenolic content of the marinades and antioxidant activity among the control (wine) and the various treatments was also performed using the same approach (ANOVA—Dunnett’s test). At all times, the significance level was 0.05. All analyses were performed using SPSS v25 (IBM Corp, Armonk, NY, USA).

## 3. Results and Discussion

### 3.1. Microbiological Analyses

**Figure 1:**
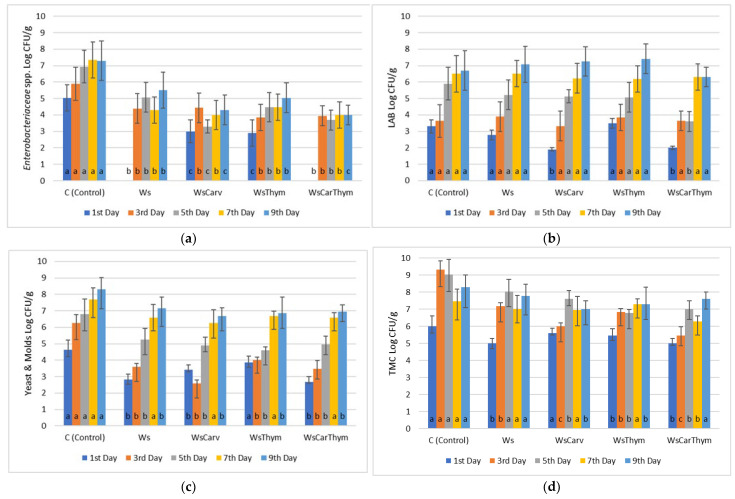
Bacterial counts of (**a**) *Enterobacteriaceae*, (**b**) Lactic Acid Bacteria (LAB), (**c**) Yeasts/Molds (Y/M) and (**d**) Total Mesophilic Count (TMC) in chicken fillets during storage (4 °C/9 days) treated with carvacrol and thymol and their combinations. ANOVA with Dunnett’s *post hoc* multiple comparison procedure was used to test for any differences between each treatment (day by day) against the control. Similar letters over bars indicate no statistical differences (*p* > 0.05). The abbreviations are: Ws: Wine-salt, WsCarv: Wine-salt-Carvacrol, WsThym: Wine-salt-Thymol, WsCarThym: Wine-Salt-Carvacrol-Thymol.

**Figure 2:**
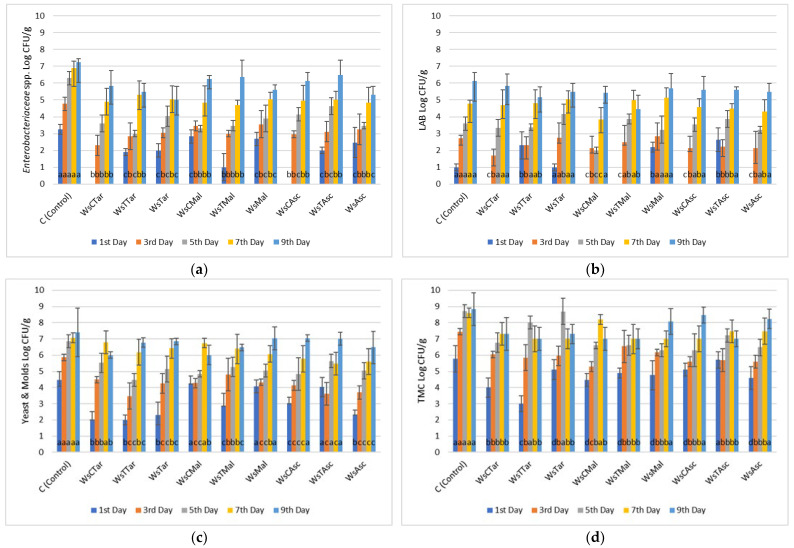
Bacterial counts of (**a**) Enterobacteriaceae, (**b**) Lactic Acid Bacteria (LAB), (**c**) Yeasts/Molds (Y/M) and (**d**) Total Mesophilic Count (TMC) in chicken fillets during storage (4 °C/9 days) treated with various combinations of carvacrol-thymol and tartaric-malic-ascorbic acid (0,1% *w*/*v*). ANOVA with Dunnett’s *post hoc* multiple comparison procedure was used to test for any differences between each treatment (day by day) against the control. Similar letters over bars indicate no statistical differences (*p* > 0.05). The abbreviations stand for: WsCTar: Wine-salt-Carvacrol-Tartaric acid, WsTTar: Wine-salt-Thymol-Tartaric acid, WsTar: Wine-salt-Tartaric acid, WsCMal: Wine-salt-carvacrol-malic acid, WsTMal: Wine-salt-Thymol-malic acid, WsMal: Wine-salt-malic acid, WsCAsc: Wine-salt-Carvacrol-Ascorbic acid, WsTAsc: Wine-salt-Thymol- Ascorbic acid, WsAsc: Wine-salt-Ascorbic acid.

*Enterobacteriaceae* count of chicken fillets was maintained at its lowest values when both carvacrol and thymol were added to the marinade, reaching a value of 4.00 ± 0.3 Log CFU/g by the 9th day of refrigerated storage. Control meat samples by the same day exhibited 7.30 ± 0.8 Log CFU/g (Figure 1). Similar findings were recorded when ascorbic acid (5.30 ± 0.7 Log CFU/g) and tartaric acid (5.00 ± 0.3 Log CFU/g) were applied in the marinade by the end of the 9th day of storage in comparison to control chicken samples (7.25 ± 0.2 Log CFU/g) for the same period (Figure 2).

**Figure 3:**
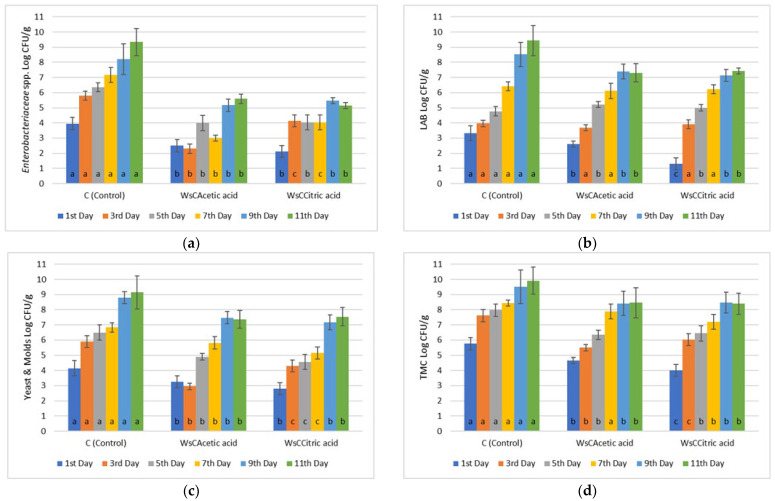
Bacterial counts of (**a**) *Enterobacteriaceae*, (**b**) Lactic Acid Bacteria (LAB), (**c**) Yeasts/Molds (Y/M), and (**d**) Total Mesophilic Count (TMC) in chicken fillets during storage (4 °C/11 days) treated with organic acids. ANOVA with Dunnett’s *post hoc* multiple comparison procedure was used to test for any differences between each treatment (day by day) against the control. Similar letters over bars indicate no statistical differences (*p* > 0.05). The abbreviations are WsCAcetic acid: Wine-salt-Carvacrol-Acetic acid, WsCCitric acid: Wine-salt-Carvacrol-Citric acid.

The application of carvacrol—acetic acid and carvacrol—citric acid provided even more promising results since a count value of 5.59 ± 0.3 Log CFU/g for the former and 5.14 ± 0.2 Log CFU/g for the latter by the 11th day were recorded (Figure 3). Chicken control samples by the 11th day of refrigerated storage exhibited a microbial count of 9.34 ± 0.3 Log CFU/g. At this point, it should be stated that the use of acetic and citric acid in marinades provided a 4 day shelf extension to chicken fillets. The addition of red wine also contributed to this effect, since such wines have slightly stronger antimicrobial effects than white ones [31].

**Figure 4:**
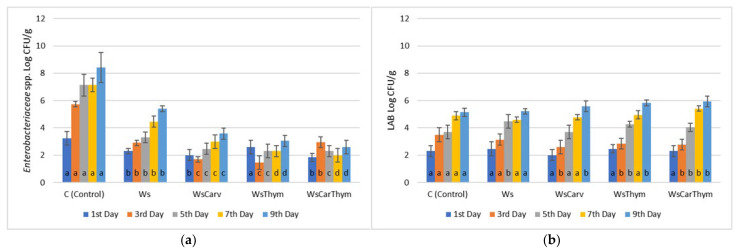

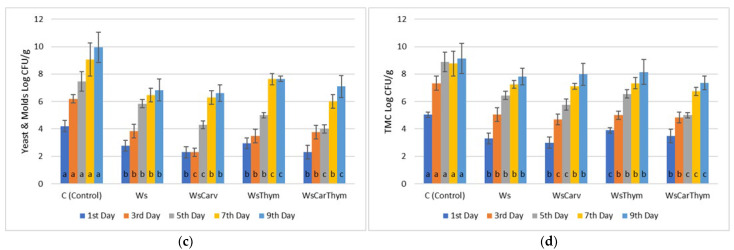
Bacterial counts of (**a**) *Enterobacteriaceae*, (**b**) Lactic Acid Bacteria (LAB), (**c**) Yeasts/Molds (Y/M), and (**d**) Total Mesophilic Count (TMC) in beef fillets during storage (4 °C/9 days) treated with carvacrol and thymol and their combinations. ANOVA with Dunnett’s *post hoc* multiple comparison procedure was used to test for any differences between each treatment (day by day) against the control. Similar letters over bars indicate no statistical differences (*p* > 0.05). The abbreviations are Ws: Wine-salt, WsCarv: Wine-salt-Carvacrol, WsThym: Wine-salt-Thymol, WsCarThym: Wine-Salt-Carvacrol-Thymol.

**Figure 5:**
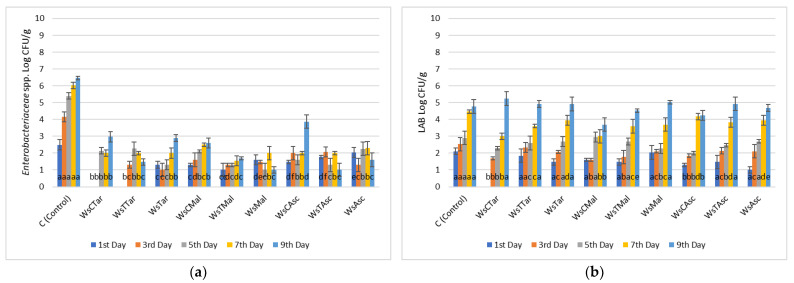

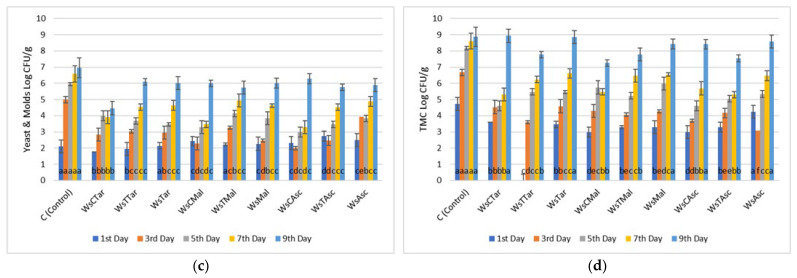
Bacterial counts of (**a**) Enterobacteriaceae, (**b**) Lactic Acid Bacteria (LAB), (**c**) Yeasts/Molds (Y/M) and (**d**) Total Mesophilic Count (TMC) in beef fillets during storage (4 °C/9 days) treated with various combinations of carvacrol-thymol and tartaric-malic-ascorbic acid (0,1% *w*/*v*). ANOVA with Dunnett’s *post hoc* multiple comparison procedure was used to test for any differences between each treatment (day by day) against the control. Similar letters over bars indicate no statistical differences (*p* > 0.05). The abbreviations stand for: WsCTar: Wine-salt-Carvacrol-Tartaric acid, WsTTar: Wine-salt-Thymol-Tartaric acid, WsTar: Wine-salt-Tartaric acid, WsCMal: Wine-salt-carvacrol-malic acid, WsTMal: Wine-salt-Thymol-malic acid, WsMal: Wine-salt-malic acid, WsCAsc: Wine-salt-Carvacrol-Ascorbic acid, WsTAsc: Wine-salt-Thymol- Ascorbic acid, WsAsc: Wine-salt-Ascorbic acid.

**Figure 6:**
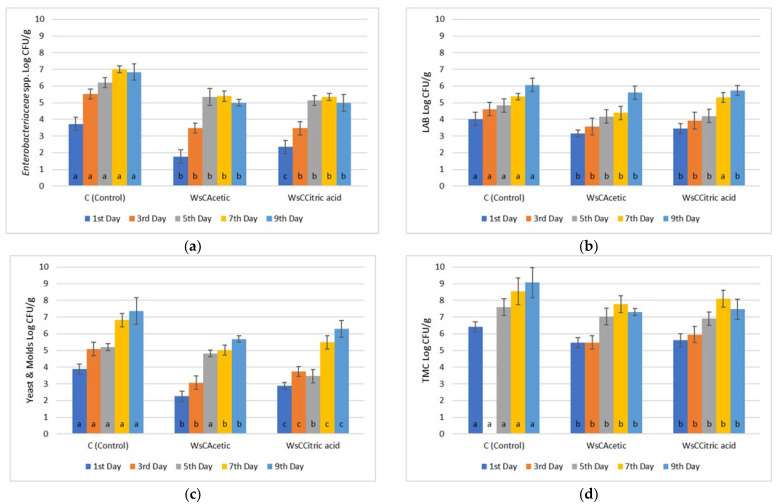
Bacterial counts of (**a**) *Enterobacteriaceae*, (**b**) Lactic Acid Bacteria (LAB), (**c**) Yeasts/Molds (Y/M) and (**d**) Total Mesophilic Count (TMC) in beef fillets during storage (4 °C/9 days) treated with organic acids. ANOVA with Dunnett’s *post hoc* multiple comparison procedure was used to test for any differences between each treatment (day by day) against the control. Similar letters over bars indicate no statistical differences (*p* > 0.05). The abbreviations are WsCAcetic acid: Wine-salt-Carvacrol-Acetic acid, WsCCitric acid: Wine-sal-Carvacrol-Citric acid.

For beef fillets, the total image was even more encouraging than for *Enterobacteriaceaeae* inhibition (Figure 4). The combined addition of carvacrol and thymol retained a microbial count of 2.58 ± 0.2 Log CFU/g during the 9th day of refrigerated storage. Tartaric, malic, and ascorbic acid reduced *Enterobacteriaceaeae* count under 3 Log CFU/g in all treatments until the 9th day of storage, compared to the 6.47 ± 0.3 Log CFU/g value of beef control samples during the 9th day (Figure 5 and Figure 6). Typical organic acids of wine, such as malic and tartaric acids, are known to exhibit inhibitory effects against several *Enteobacteriaceae* species [32,33], in combination with synergistic effects between ethanol, organic acids, and low pH [31]. In another study, Just and Daeschel [34] proved that *Enteobacteriaceae* may survive much longer in grape juice than in red wine, despite the same pH. This fact indicates the significance of organic acids and metabolites that form during fermentation [31].

Arcanjo et al. [35] demonstrated that Isabel wine inhibited *Enterobacteriaceae* growth because of its phenolic composition and its high organic content, while the presence of sugars in cabernet and Tempranillo wines enhanced the growth of LAB in wine-marinated beef. Our results verify these observations since the LAB population varied almost in the same range of counts as the control meat samples for both chicken and beef fillets. In some cases (wine-salt, wine-salt-carvacrol, and wine-salt-thymol), the LAB count for each treatment was higher than that of the control samples on the respective day, indicating that wine itself may contain an endogenous LAB population. Furthermore, the yeast and mold populations retained their growth during the refrigerated storage period. In all treatments, for both chicken and beef fillets, the population was lower compared to that of the control samples.

More specifically, for chicken fillets, the most effective treatments were marinades with wine-salt-carvacrol-tartaric acid and wine-salt-carvacrol-malic acid (6.00 ± 0.7 Log CFU/g during the 9th day). As for beef fillet, the treatment with marinade with wine-salt-carvacrol-tartaric acid exhibited the lowest count values (4.47 ± 0.5 Log CFU/g during the 9th day). In our previous study concerning pork fillets, the application of wine-salt-Oregano Essential Oil (OEO)-pomegranate extract resulted in 5.61 ± 0.38 Log CFU/g during the 7th day of refrigerated storage [36]. Therefore, it can be concluded that the application of a single bioactive compound, in combination with organic acids, controls the yeasts/molds population in marinated meat samples in a more effective way. A possible explanation for this observation may be the synergistic interactions between the organic acids of the wine (malic-citric acid) or those added (tartaric acid) and carvacrol (Figure 5 and Figure 6). It has been suggested that hydrophobic compounds like carvacrol and thymol could alter the permeability of microbial membranes for ions, changing internal pH, and affecting cell activity [37]. Furthermore, Total Mesophilic Counts (TMC) in chicken fillets were retained under the limit of 7 Log CFU/g until the 9th day of storage with the treatments of wine-salt-carvacrol, wine-salt-thymol-tartaric acid, wine-salt-carvacrol-malic acid, wine-salt-thymol-malic acid, and wine-salt-thymol-ascorbic acid. In the case of beef fillets, treatment with wine-salt-carvacrol-thymol (7.35 ± 0.45 Log CFU/g), wine-salt-carvacrol-malic acid (7.25 ± 0.5 Log CFU/g), and wine-salt-carvacrol-acetic acid (7.30 ± 0.45 Log CFU/g) by the 9th day of refrigerated storage, while beef control samples exceeded the 9 Log CFU/g value by the same period of storage. Similar findings have also been reported by other scientists. Kargiotou et al. [38] stated that marinating beef in red wine may extend the shelf-life by reducing the total live count which is responsible for spoilage in raw meat. The synergy between phenolic compounds has been clearly observed in the literature. Several authors [39,40,41] have reported the antimicrobial activity of marinades regardless of the type of marination. Especially, the combination of organic acids, ethanol, and sodium chloride may inhibit the growth of several foodborne pathogens [25,42]. Citric and acetic acids demonstrate antimicrobial activity, mainly in their undissociated form, mostly by invading cell membranes and acidifying cytoplasm, or even by destabilizing the cell’s outer membrane [43]. Another mode of antimicrobial action of weak organic acids involves other hydrophobic molecules entering the membrane, as in our case. The rate of their action against foodborne pathogens depends on their concentration, pH, and temperature [44]. In contrast with the observations of others researchers [39,40], we did not observe any significant difference between the antimicrobial activity of acetic and citric acid.

Elliopoulos and Moellering [45] stated that synergy between compounds is also reported when the combined antimicrobials demonstrate an additional one-log reduction compared to the sum of the lethal effects of each preservative. Based on this definition, we may state that with the same concentration (1000 mg/L) of bioactive compounds (carvacrol-thymol) and a low concentration of organic acids (0.1% *w*/*v*), we observed statistically lower microbial counts in all microbial groups examined and simultaneously, 2 or even 4 days of life extension in comparison with our previous work in which OEO and TEO were applied in pork fillets [36]. It is supposed that the high levels of fat and lower water content in food matrices may hamper the progress of antimicrobial agents in the final target in the cell; if the essential oil or bioactive substance dissolves in the lipid phase, then there will be less quantity available to act against foodborne pathogens in the aqueous phase [46]. It is common knowledge that a greater amount of essential oils is needed in order to achieve the same effect in food systems [47].

Overall, we may infer that in chicken meat, wine-based marinates with mixtures of carvacrol and thymol were capable of lowering the initial (1st day) and final (9th day) bacterial counts by 28% when compared with the control samples (marinades of wine only). This was profound, mostly with *Enterobacteriaceae* and yeasts and molds, which are the most important since these microorganisms are both spoilage and pathogens. Chicken marinades with mixtures of carvacrol—thymol and tartaric—malic—ascorbic acid were also effective against *Enterobacteriaceae* and yeasts and molds, but only up to the 7th day of observation.

In beef meat, both wine-based marinates with mixtures of carvacrol—thymol and mixtures of tartaric-malic-ascorbic acid were equally effective against *Enterobacteriaceae*. During the entire period of observation in those treatments, the microbial load ranged between 2 and 3 Log cfu/g for carvacrol-thymol mixtures and 1–1.5 Log cfu/g for tartaric-malic-ascorbic mixtures, while in control samples, it reached up to 8.44 ± 1.1 Log cfu/g and 6.47 ± 0.4 Log fu/g, respectively.

Chicken meat, in comparison to beef, more likely allows for a higher rate of microbial multiplication, mostly due to its higher protein content [9]. In our study, both types of meat kept under similar storage conditions (4 °C/9 days) exhibited lower microbial growth, particularly with *Enterobacteriaceae,* when treated with wine-based carvacrol—thymol marinades. This antimicrobial action was more obvious in beef samples.

### 3.2. Total Phenolic Content (TPC)

The total phenolic content of the marinades applied was determined, and the results are presented in Table 1.

From the above analytical results, it is obvious that the marinade that contained the combination of carvacrol and acetic acid had the highest TPC value (1.735 ± 0.17 mg GA/mL marinade), followed by the combination of carvacrol and ascorbic acid (1.488 ± 0.15 mg GA/mL marinade). The addition of ascorbic acid resulted in high TPC when combined with thymol (1.144 ± 0.12 mg GA/mL marinade). Above the limit of 1 mg GA/mL marinade varied the contained carvacrol (1.082 ± 0.25 mg GA/mL marinade) and the marinade which contained carvacrol and thymol (1.010 ± 0.21 mg GA/mL marinade). The marinades containing thymol/tartaric acid and only tartaric acid exhibited the lowest TPC values (0.695 ± 0.10 mg GA/mL marinade and 0.646 ± 0.08 mg GA/mL marinade, respectively). Our findings are in accordance with Rathee et al. [48], who concluded that the total antioxidant potential of plant materials, such as herbs, spices, fruits, and vegetables, is connected with ascorbic acid (vitamin C), α-tocopherol (vitamin E), β-carotene, flavonoids and phenolic compounds. This conclusion may explain why the combination of carvacrol and thymol with ascorbic acid resulted in the highest TPC values among the marinades examined.

### 3.3. Antioxidant Activity (DPPH and ABTS Radical Scavenging Methods)

The antioxidant activity of the wine-based marinades was examined using the DPPH and ABTS radical scavenging methods, and the results are presented in Table 2.

Results revealed that the marinades which contained only wine (254.291 ± 8.52 μg Trolox/mL of marinade), wine/salt/thymol (245.125 ± 8.95 μg Trolox/mL marinade), wine/salt (240.958 ± 7.77 Trolox/mL marinade), wine/salt/carvacrol/acetic acid (190.958 ± 4.32 μg Trolox/mL marinade) and wine/salt/carvacrol/ascorbic acid (205.125 ± 7.85 μg Trolox/mL marinade) through DPPH method. These findings are in agreement with previous studies in the literature, which mentioned the high antioxidant activity of grape seed extract [49,50]. Additionally, Rohod et al. [51] demonstrated that rosemary and oregano have antioxidant and antimicrobial activities similar to those of synthetic antioxidants, such as BHT, in marinated chicken breast. It is widely accepted that natural antioxidants, such as phenolic compounds and ascorbic acid, may contribute to inhibiting cyclization and oxidation reactions by quenching or scavenging free radicals, thereby ameliorating shelf-life and safety of meat products [52].

According to Okpala et al. [9], the DPPH radical scavenging activity of different marinades containing cranberry pomace, grape pomace with either African spices, or an industrial marinade pickle could be applied to chicken breast meat. The results revealed minimum DPPH values at 225.27 μg Trolox/mL marinade and maximum DPPH values at 375.4 μg Trolox/mL for the industrial marinade.

As for ABTS assay, our findings demonstrated that all the marinades with only wine, wine-salt, wine-salt-carvacrol, wine-salt-thymol, wine-salt-carvacrol-thymol, wine-salt-carvacrol-tartaric acid, wine salt-thymol-tartaric acid, and wine-salt-malic acid varied around the value of 468 μg Trolox/mL marinade, followed by the marinade with wine-salt-carvacrol-ascorbic acid (464.86 μg Trolox/mL marinade) and wine-salt-thymol-ascorbic acid (459.14 μg Trolox/mL marinade). All other combinations decreased to 41 μg Trolox/mL marinade, up to 86 μg Trolox/mL marinade. The respective ABTS values in the Okpala et al. [9] study varied from 455.5 μg Trolox/mL marinade to 630.756 μg Trolox/mL marinade.

As it is known from the literature, wine, mainly red, is frequently used as a marination base. Its complex composition consists of more than 600 substances, water, ethanol, organic acids, sugars, pigments, and polyphenols, which are responsible for a significant pH reduction and antioxidant activity [53].

The presence of organic acids, such as tartaric, malic, succinic, lactic, and acetic acid, formulates its pH somewhere between 3.0–3.6, and its application may inhibit microbial growth and delay the oxidation of lipids and proteins [54]. The varietal diversity of wine may influence the antioxidant capacity and quality parameters of marinated meat. For example, Arcanjo et al. [35] stated that cabernet and Tempranillo wines are more effective against lipid oxidation than Isabel wine because of their rich procyanidin content.

A positive correlation between TPC and DPPH and ABTS antioxidant activities is not always observed. In most cases, when the TPC values increase, the respective DPPH and ABTS values increase as well due to the fact that phenolic compounds easily accept electrons of hydrogen radicals and consequently become stable diamagnetic molecules [55]. In some other cases, this happens above a specific threshold value, and finally, in a few cases, this correlation is reversed accordingly due to the fact that some enzymes, for example, are not activated [56].

### 3.4. pH Determination

**Figure 7:**
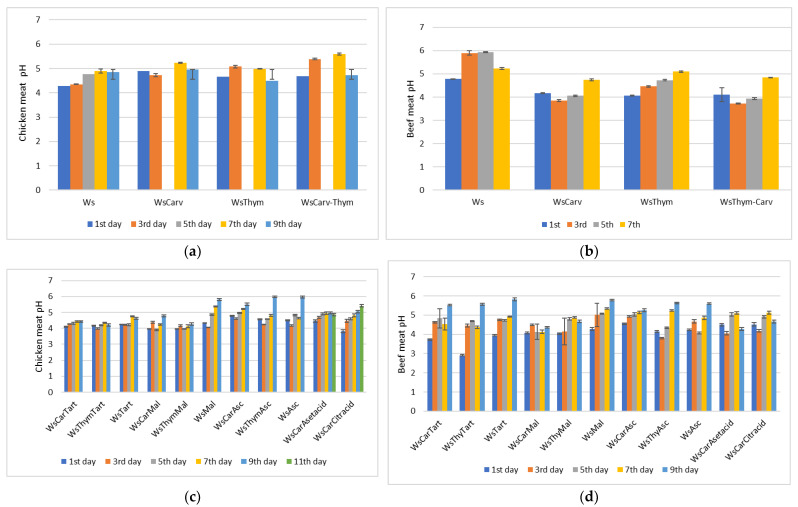
pH values of marinades applied to meat fillets (chicken and beef) during storage (4 °C) treated with carvacrol and thymol (**a**,**b**) and their combinations with tartaric/malic/ascorbic acid and acetic/citric acid (**c**,**d**). The abbreviations stand for: WsCTar: Wine-salt-Carvacrol-Tartaric acid, WsTTar: Wine-salt-Thymol-Tartaric acid, WsTar: Wine-salt-Tartaric acid, WsCMal: Wine-salt-carvacrol-malic acid, WsTMal: Wine-salt-Thymol-malic acid, WsMal: Wine-salt-malic acid, WsCAsc: Wine-salt-Carvacrol-Ascorbic acid, WsTAsc: Wine-salt-Thymol- Ascorbic acid, WsAsc: Wine-salt-Ascorbic acid.

The pH values of marinades applied in meat fillets (chicken and beef) during storage (4^ο^C) treated with carvacrol and thymol and their combinations with tartaric/malic/ascorbic acid and acetic/citric acid are presented in Figure 7.

The pH values of marinades for both chicken beef fillets followed the same trend. During the 1st day of marination, the pH values varied from 3.82 to 4.89 for chicken samples and from 2.92 to 4.55 for beef samples. The respective pH values for the control samples were 4.29 and 4.76.

The pH of the marinades increased throughout storage. It is noteworthy to state that regarding microbial counts, the lowest values were not recorded for the marinades with the most acidic pH values. This observation highlights the fact that the mode of antimicrobial activity of the combinations of carvacrol-thymol with organic acids was not mediated by the created acidic conditions. Nevertheless, it is common knowledge that at low pH, the molecules of thymol and carvacrol are mostly dissociated and more hydrophobic, and as a result, they are able to bind better to the hydrophobic regions of the membrane proteins [57].

## 4. Conclusions

The present study examined the antimicrobial activity of wine-based marinades containing carvacrol and/or thymol, alone or in combination with organic acids (ascorbic, malic, tartaric, acetic, and citric acid). The food systems used were marinated chicken and beef fillets, and the microbial groups tested were *Enterobacteriaceae*, Total Mesophilic Bacteria, Yeasts/molds, and lactic acid bacteria (LAB). Our findings demonstrated that both types of meat kept under similar storage conditions (4 °C/11 days) exhibited lower microbial growth, particularly with *Enterobacteriaceae,* when treated with wine-based carvacrol—thymol marinades and may extend their shelf-life up to 4 days. This antimicrobial action was more profound in beef samples. Marinades containing thymol and/or carvacrol in combination with ascorbic and acetic acids had the highest TPC and antioxidant activity values, and both methods were applied to both chicken and beef samples. Meanwhile, the pH values exhibited an ascending trend during storage, leading to the conclusion that acidic conditions are not the reason for the observed antimicrobial activity.

On this basis, the combination of thymol and carvacrol with organic acids could replace synthetic antioxidants, which are widely used in meat and meat product preservation as antimicrobials and antioxidants without affecting the odor and flavor of meat samples like essential oils do. Further investigations should be conducted in order for more alternative combinations to be determined and their possible sensorial attributes.

## Figures and Tables

**Table 1:** The Total Phenolic Content of the wine-based marinades and their combinations with carvacrol/thymol and tartaric/malic/ascorbic/acetic/citric acid, expressed as mg/mL of Gallic Acid.

MARINADES	TPC (mg GA/mL Marinade)
1. Wine	0.83 ^c^ ± 0.11
2. Wine Salt	0.77 ^d^ ± 0.05
3. Wine Salt Carvacrol	1.08 ± 0.25
4. Wine Salt Thymol	0.76 ^d^ ± 0.05
5. Wine Salt Carvacrol/Thymol	1.01 ^c^ ± 0.11
6. Wine Salt Carvacrol Tartaric acid	0.79 ^c^ ± 0.03
7. Wine Salt Thymol Tartaric acid	0.69 ^d^ ± 0.10
8. Wine Salt Tartaric acid	0.65 ^d^ ± 0.08
9. Wine Salt Carvacrol Malic acid	0.94 ^c^ ± 0.11
10. Wine Salt Thymol Malic acid	0.78 ^c^ ± 0.05
11. Wine Malic acid	0.83 ^c^ ± 0.12
12. Wine Salt Carvacrol Ascorbic acid	1.49 ^b^ ±0.15
13. Wine Salt Thymol Ascorbic acid	1.14 ^c^ ± 0.12
14. Wine salt Ascorbic acid	0.96 ^a^ ±0.13
15. Wine Salt Carvacrol Acetic acid	1.74 ^a^ ±0.17
16. Wine Salt Citric acid	0.89 ^a^ ±0.14

Different superscript letters in the columns indicate statistically significant differences (ANOVA; Duncan’s multiple range test; *p* < 0.05).

**Table 2:** The antioxidant activity of the wine-based marinades with carvacrol/thymol and their combinations with tartaric/malic/ascorbic/acetic/citric acid was determined using DPPH and ABTS radical scavenging methods and expressed as μg Trolox/mL marinade.

MARINADES	ABTS	DPPH
1.Wine	468.85 ^a^ ± 10.23	254.29 ^a^ ± 8.52
2. Wine Salt	468.85 ^a^ ±11.45	240.96 ^a^ ± 7.77
3. Wine Salt Carvacrol	468.82 ^a^ ±10.78	148.04 ^d^ ± 5.21
4. Wine Salt Thymol	468.91 ^a^ ±12.41	245.13 ^a^ ± 8.95
5. Wine Salt Carvacrol/Thymol	468.73 ^a^ ±10.48	168.46 ^c^ ± 5.32
6. Wine Salt Carvacrol Tartaric acid	468.72 ^a^ ±10.32	137.21 ^d^ ± 4.51
7. Wine Salt Thymol Tartaric acid	468.77 ^a^ ±11.52	145.13 ^d^ ± 6.23
8. Wine Salt Tartaric acid	50.09 ^d^ ± 2.58	89.71 ^e^ ± 3.57
9. Wine Salt Carvacrol Malic acid	68.67 ^c^ ± 3.33	15.54 ^h^ ± 1.54
10. Wine Salt Thymol Malic acid	86.76 ^b^ ± 4.21	27.21 ^g^ ± 2.22
11. Wine Malic acid	70.09 ^c^ ± 2.24	73.46 ^f^ ± 3.66
12. Wine Salt Carvacrol Ascorbic acid	468.71 ^a^ ± 10.51	205.13 ^b^ ± 7.85
13. Wine Salt Thymol Ascorbic acid	464.86 ^a^ ± 10.56	224.29 ^a^ ±8.35
14. Wine salt Ascorbic acid	459.14 ^a^ ± 11.25	191.79 ^b^ ± 6.52
15. Wine Salt Carvacrol Acetic acid	66.76 ^c^ ± 2.21	190.96 ^b^ ± 9.32
16. Wine Salt Citric acid	41.80 ^e^ ± 1.11	224.71 ^a^ ± 7.78

Different superscript letters in the columns indicate statistically significant differences (ANOVA; Duncan’s multiple range test; *p* < 0.05).

## Data Availability

The data presented in this study are available upon request from the corresponding author.

## Supplementary Materials

The following supporting information can be downloaded at: https://www.mdpi.com/article/10.3390/microorganisms13010182/s1, Table S1: Number of samples and respective quantities for each microbiological and physicochemical analysis with all marinade treatments applied for chicken and beef meat samples.

## References

[1] WHO (2024). Foodborne Disease Burden Epidemiology Reference Group for 2021–2024: Second Meeting Report, 19 October–2 November 2021.

[2] Kalogianni A.I., Lazou T., Bossis I., Gelasakis A.I. (2020). Natural phenolic compounds for the control of oxidation, bacterial spoilage, and foodborne pathogens in meat. Foods.

[3] Rahman M., Hashem M., Azad M., Choudhury M., Bhuiyan M. (2023). Techniques of meat preservation—A review. Meat Res..

[4] Zhang H., Sun C., Han W., Zhang J., Hou J. (2018). Analysis of the monitoring status of residual nitrite in meat products in China from 2000 to 2011. Meat Sci..

[5] Zhou G.H., Xu X.L., Liu Y. (2010). Preservation technologies for fresh meat—A review. Meat Sci..

[6] Addis M. (2015). Major Causes of Meat Spoilage and Preservation Techniques: A Review. Changes.

[7] Šojić B., Pavlić B., Ikonić P., Tomović V., Ikonić B., Zeković Z., Kocić-Tanackov S., Jokanović M., Škaljac S., Ivić M. (2019). Coriander essential oil as natural food additive improves quality and safety of cooked pork sausages with different nitrite levels. Meat Sci..

[8] Adamski M., Kuzniacka J., Milczewska N. (2017). Preferences of consumers for choosing poultry meat. Pol. J. Nat. Sci..

[9] Okpala C.O.R., Juchniewicz S., Leicht K., Korzeniowska M., Guiné R.P. (2022). Antioxidant, organoleptic and physicochemical changes in different marinated oven-grilled chicken breast meat. Foods.

[10] De Liu X., Jayasena D.D., Jung Y., Jung S., Kang B.S., Heo K.N., Lee J.H., Jo C. (2012). Differential Proteome Analysis of Breast and Thigh Muscles between Korean Native Chickens and Commercial Broilers. Asian-Australas. J. Anim. Sci..

[11] Juárez M., Lam S., Bohrer B.M., Dugan M.E.R., Vahmani P., Aalhus J., Juárez A., López-Campos O., Prieto N., Segura J. (2017). Nutrient density and nutritional value of meat products and non-meat foods high in protein. Trends Food Sci. Technol..

[12] Ahmad R.S., Imran A., Hussain M.B. (2018). Nutritional composition of meat. Meat Sci. Nutr..

[13] Bhat Z.F., Morton J.D., Mason S.L., Bekhit A.E.D.A. (2018). Applied and Emerging Methods for Meat Tenderization: A Comparative Perspective. Compr. Rev. Food Sci. Food Saf..

[14] Scollan N.D., Price E.M., Morgan S.A., Huws S.A., Shingfield K.J. (2017). Can we improve the nutritional quality of meat?. Proc. Nutr. Soc..

[15] Ehsanur Rahman S.M., Islam S., Pan J., Kong D., Xi Q., Du Q., Yang Y., Wang J., Oh D.-H., Han R. (2023). Marination ingredients on meat quality and safety—A review. Food Qual. Saf..

[16] Barbut S. (2002). Poultry Products Processing: An Industry Guide.

[17] Panić M., Stojković M.R., Kraljić K., Škevin D., Redovniković I.R., Srček V.G., Radošević K. (2019). Ready-to-use green polyphenolic extracts from food by-products. Food Chem..

[18] Martucci J.F., Gendeb L.B., Neiraa L.M., Ruseckaite R.A. (2015). Oregano and lavender essential oils as antioxidant and antimicrobial additives of biogenic gelatin films. Ind. Crops Prod..

[19] Hossain F., Follett P., Dang Vu K., Harich M., Salmieri S., Lacroix M. (2016). Evidence for synergistic activity of plant-derived essential oils against fungal pathogens of food. Food Microbiol..

[20] Karam L., Chehab R., Osaili T.M., Savvaidis I.N. (2020). Antimicrobial effect of thymol and carvacrol added to a vinegar-based marinade for controlling spoilage of marinated beef (Shawarma) stored in air or vacuum packaging. Int. J. Food Microbiol..

[21] Hajibonabi A., Yekani M., Sharifi S., Nahad J.S., Dizaj S.M., Memar M.Y. (2023). Antimicrobial activity of nanoformulations of carvacrol and thymol: New trend and applications. OpenNano.

[22] Rúa J., Del Valle P., de Arriaga D., Fernández-Álvarez L., García-Armesto M.R. (2019). Combination of carvacrol and thymol: Antimicrobial activity against *Staphylococcus aureus* and antioxidant activity. Food. Path. Dis..

[23] Dimitrijević M., Stankov Jovanović V., Cvetković J., Mitić M., Petrović G., Đorđević A., Mitić V. (2017). Phenolics, antioxidant potentials, and antimicrobial activities of six wild Boletaceae mushrooms. Anal. Lett..

[24] Naveena B.M., Muthukumar M., Sen A.R., Babji Y., Murthy T.R.K. (2006). Improvement of shelf-life of buffalo meat using lactic acid, clove oil and vitamin C during retail display. Meat Sci..

[25] Lytou A.E., Tzortzinis K., Skandamis P.N., Nychas G.J.E., Panagou E.Z. (2019). Investigating the influence of organic acid marinades, storage temperature and time on the survival/inactivation interface of Salmonella on chicken breast fillets. Int. J. Food Microbiol..

[26] Meneses R., Teixeira P. (2022). Marination as a hurdle to microbial pathogens and spoilers in poultry meat products: A brief review. Appl. Sci..

[27] Marshall D.L., Kim C.R. (1996). Microbiological and sensory analyses of refrigerated catfish fillets treated with acetic and lactic acids. J. Food Qual..

[28] Huang D., Ou B., Prior R.L. (2005). The chemistry behind antioxidant capacity assays. J. Agric. Food Chem..

[29] Re R., Pellegrini N., Proteggente A., Pannala A., Yang M., Rice-Evans C. (1999). Antioxidant activity applying an improved ABTS radical cation decolorization assay. Free Radic. Biol. Med..

[30] Frankel E.N., Meyer A.S. (2000). The problems of using one-dimensional methods to evaluate multifunctional food and biological antioxidants. J. Sci. Food Agric..

[31] Møretrø T., Daeschel M.A. (2004). Wine is bactericidal to foodborne pathogens. J. Food Sci..

[32] Rhoades J., Kargiotou C., Katsanidis E., Koutsoumanis K.P. (2013). Use of marination for controlling Salmonella enterica and Listeria monocytogenes in raw beef. Food Microbiol..

[33] Likotrafiti E., Tuohy K.M., Gibson G.R., Rastall R.A. (2013). Development of antimicrobial synbiotics using potentially-probiotic faecal isolates of *Lactobacillus fermentum* and Bifidobacterium longum. Anaerobe.

[34] Just J.R., Daeschel M.A. (2003). Antimicrobial effects of wine on *Escherichia coli* O157: H7 and Salmonella typhimurium in a model stomach system. J. Food Sci..

[35] Arcanjo N.M.O., Morcuende D., Andrade M.J., Padilla P., Madruga M.S., Estévez M. (2019). Bioactivities of wine components on marinated beef during aging. J. Funct. Foods.

[36] Mantzourani I., Daoutidou M., Nikolaou A., Kourkoutas Y., Alexopoulos A., Tzavellas I., Dasenaki M., Thomaidis N., Plessas S. (2023). Microbiological stability and sensorial valorization of thyme and oregano essential oils alone or combined with ethanolic pomegranate extracts in wine marinated pork meat. Int. J. Food Microbiol..

[37] Papadopoulou A., Frazier R.A. (2004). Characterization of protein–polyphenol interactions. Trends Food Sci. Technol..

[38] Kargiotou C., Katsanidis E., Rhoades J., Kontominas M., Koutsoumanis K. (2011). Efficacies of soy sauce and wine base marinades for controlling spoilage of raw beef. Food Microbiol..

[39] Nisiotou A., Chorianopoulos N.G., Gounadaki A., Panagou E.Z., Nychas G.J. (2013). Effect of wine-based marinades on the behavior of *Salmonella typhimurium* and background flora in beef fillets. Int. J. Food Microbiol..

[40] Bolumar T., Andersen M.L., Orlien V. (2014). Mechanisms of radical formation in beef and chicken meat during high pressure processing evaluated by electron spin resonance detection and the addition of antioxidants. Food Chem..

[41] Vaquero M.J.R., Alberto M.R., de Nadra M.C.M. (2007). Influence of phenolic compounds from wines on the growth of *Listeria monocytogenes*. Food Control.

[42] Friedman M. (2007). Overview of antibacterial, antitoxin, antiviral, and antifungal activities of tea flavonoids and teas. Mol. Nutr. Food Res..

[43] Mani-López E., García H.S., López-Malo A. (2012). Organic acids as antimicrobials to control Salmonella in meat and poultry products. Food Res. Int..

[44] Taylor T.M., Doores S.X. (2020). Organic acids. Antimicrobials in Food.

[45] Eliopoulos G.M., Moellering R.C. (1982). Antibiotic synergism and antimicrobial combinations in clinical infections george. Rev. Infect. Dis..

[46] Burt S. (2004). Essential oils: Their antibacterial properties and potential applications in foods—A review. Int. J. Food Microbiol..

[47] Pol I.E., Mastwijk H.C., Slump R.A., Popa M.E., Smid E.J. (2001). Influence of food matrix on inactivation of *Bacillus cereus* by combinations of nisin, pulsed electric field treatment, and carvacrol. J. Food Protect..

[48] Rathee J.S., Patro B.S., Mula S., Gamre S., Chattopadhyay S. (2006). Antioxidant activity of *Piper betel* leaf extract and its constituents. J. Agric. Food Chem..

[49] Arora P., Ansari S.H., Nazish I. (2010). Bio-functional aspects of grape seeds—A review. Int. J. Phytomed..

[50] Chedea V.S., Braicu C., Socaciu C. (2010). Antioxidant/prooxidant activity of a polyphenolic grape seed extract. Food Chem..

[51] Rohod R.V., Garcia E.R.D.M., Lara J.A.F.D. (2022). Natural extracts marination in chicken breast fillets. Ciênc. Rural.

[52] Onopiuk A., Kołodziejczak K., Szpicer A., Marcinkowska-Lesiak M., Wojtasik-Kalinowska I., Stelmasiak A., Poltorak A. (2022). The Effect of Partial Substitution of Beef Tallow on Selected Physicochemical Properties, Fatty Acid Profile and PAH Content of Grilled Beef Burgers. Foods.

[53] Birk T., Grønlund A.C., Christensen B.B., Knøchel S., Lohse K., Rosenquist H. (2010). Effect of organic acids and marination ingredients on the survival of Campylobacter jejuni on meat. J. Food Protect..

[54] Volschenk H., Van Vuuren H.J.J., Viljoen-Bloom M. (2006). Malic Acid in Wine: Origin, Function and Metabolism During Vinification. S. Afr. J. Enol. Vitic..

[55] Prakash D., Suri S., Upadhyay G., Singh B.N. (2007). Total phenol, antioxidant and free radical scavenging activities of some medicinal plants. Int. J. Food Sci. Nutr..

[56] Clarke G., Ting K.N., Wiart C., Fry J. (2013). High correlation of 2, 2-diphenyl-1-picrylhydrazyl (DPPH) radical scavenging, ferric reducing activity potential and total phenolics content indicates redundancy in use of all three assays to screen for antioxidant activity of extracts of plants from the Malaysian rainforest. Antioxidants.

[57] Coloretti F., Tabanelli G., Chiavari C., Lanciotti R., Grazia L., Gardini F., Montanari C. (2014). Effect of wine addition on microbiological characteristics, volatile molecule profiles and biogenic amine contents in fermented sausages. Meat Sci..

