# Supplementary material for: The Antimicrobial Effect of Thymol and Carvacrol in Combination with Organic Acids Against Foodborne Pathogens in Chicken and Beef Meat Fillets
Source: Microorganisms. 2025 Jan 16;13(1):182. doi: 10.3390/microorganisms13010182 (PMC11767868; doi:10.3390/microorganisms13010182)
Supplement: Supplementary file 1 [file microorganisms-13-00182-s001.zip › microorganisms-3318826-supplementary.pdf]

Table S1: Number of samples and respective quantities for each microbiological and physicochemical analysis with all marinade treatments applied for chicken and beef meat samples									
	**Microbiological**				**Physicochemical**				
Marinade	Chicken samples prepared and stored (4°C for 11 days)	Chicken samples analyzed	Beef samples prepared and stored (4°C for 11 days)	Beef samples analyzed	TPC	DPPH	ABTS	Chicken fillets marinade pH	Beef fillets marinade pH
(W) Wine	-	-	-	-	3	3	3	-	-
(Ws) Wine-salt	5x50g	18x10g	5x50g	15x10g	3	3	3	15	12
(WsCarv) Wine-salt-Carvacrol	5x50g	15x10g	5x50g	15x10g	3	3	3	15	12
(WsThym) Wine-salt-Thymol	5x50g	15x10g	5x50g	15x10g	3	3	3	15	12
(WsCar-Thym) Wine-Salt-Carvacrol-Thymol	5x50g	15x10g	5x50g	15x10g	3	3	3	15	12
(WsCTar) Wine-salt-Carvacrol-Tartaric acid	5x50g	15x10g	5x50g	15x10g	3	3	3	18	15
(WsTTar) Wine-salt-Thymol-Tartaric acid	5x50g	15x10g	5x50g	15x10g	3	3	3	18	15
(WsTar) Wine-salt-Tartaric acid	5x50g	15x10g	5x50g	15x10g	3	3	3	18	15
(WsCMal) Wine-salt-carvacrol-malic acid	5x50g	15x10g	5x50g	15x10g	3	3	3	18	15
(WsTMal) Wine-salt-Thymol-malic acid	5x50g	15x10g	5x50g	15x10g	3	3	3	18	15
(WsMal) Wine-salt-malic acid	5x50g	15x10g	5x50g	15x10g	3	3	3	18	15
(WsCAsc) Wine-salt-Carvacrol-Ascorbic acid	5x50g	15x10g	5x50g	15x10g	3	3	3	18	15

(WsTAsc) Wine-salt-Thymol- Ascorbic acid	5x50g	15x10g	5x50g	15x10g	3	3	3	18	15
(WsAsc) Wine-salt-Ascorbic acid	5x50g	15x10g	5x50g	15x10g	3	3	3	18	15
(WsCAcetic acid) Wine-salt-Carvacrol-Acetic acid	5x50g	18x10g	5x50g	15x10g	3	3	3	18	15
(WsCCitric acid) Wine-salt-Carvacrol-Citric acid	5x50g	18x10g	5x50g	15x10g	3	3	3	18	15
Total samples	90x50g	234x10g	90x50g	225x10g	48	48	48	258	213
